# Up-regulation of DcR3 in microbial toxins-stimulated HUVECs involves NF-κB signalling

**DOI:** 10.1186/s12858-018-0102-z

**Published:** 2018-12-27

**Authors:** Yanqiang Hou, Dongyu Liang, Yang Liu, Hongwei Chen, Xiaoli Lou

**Affiliations:** Department of Central Laboratory, Songjiang Hospital Affiliated First People’s Hospital, Shanghai Jiao Tong University, NO.748 Middle Zhongshan Road, Songjiang District, Shanghai, 201600 China

**Keywords:** Sepsis, HUVECs, DcR3, NF-κB

## Abstract

**Background:**

Sepsis is a severe condition characterised by the body’s systemic inflammatory response to infection. The specific sepsis-related biomarkers should be used in clinical diagnosis, therapeutic response monitoring, rational use of antibiotics, and prognosis (risk stratification), etc.

**Results:**

In this study, we investigated the expression level of Decoy Receptor 3 (DcR3) and the mechanism of high expression in sepsis patients. Septic cell model experiments were performed by treating human umbilical vein endothelial cells (HUVECs) and Jurkat cells with lipopolysaccharide (LPS), lipoteichoic acid (LTA) and zymosan, respectively. SP600125, SB203580 and ammonium pyrrolidinedithiocarbamate (PDTC) were used to inhibit JNK1/2, p38MAPK and NF-κB signalling pathways in septic cell model, respectively. These results showed that DcR3 levels were higher in sepsis group than control. DcR3 mRNA and protein levels in HUVECs were increased following treatment with LPS, LTA and zymosan, and also increased in Jurkat cells treated by LPS, but not by LTA or zymosan. When HUVECs were treated with the NF-κB inhibitor PDTC, DcR3 expression was decreased compared with controls. However, SP600125 and SB203580 had no effect on DcR3 mRNA or protein levels.

**Conclusions:**

The results indicated that DcR3 secretion proceeded through the NF-κB signalling pathway in HUVECs.

## Background

Sepsis is a condition characterised by the body’s inflammatory response to infection, and it is diagnosed where there is evidence of systemic inflammation in addition to confirmed or suspected bloodstream infection. The yearly incidence of sepsis is 300 cases per 100,000 and has been increasing years. This disease accounts for 2% of hospital admissions, roughly 9% of patients with sepsis progress to severe sepsis and 3% of those with severe sepsis experience septic shock, the most severe complication of sepsis, which is a deadly disease [1, 2]. In the last decade, a series of initiatives were implemented that aim not only to improve the understanding of sepsis and the clarity of concepts related to this condition but also to reduce morbidity and mortality due to sepsis through earlier diagnosis and initiation of antibiotic therapy.

The known presence of specific biomarkers during the response to an infectious insult makes possible the potential clinical use of such biomarkers in screening, diagnosis, prognosis (risk stratification), therapeutic response monitoring, and rational use of antibiotics (determination of adequate treatment length, for example). Procalcitonin (PCT), C-reactive protein (CRP), Interleukin 6 (IL-6), Interleukin 8 (IL-8), Interleukin 18 (IL-18), Human neutrophil gelatinase (NGAL) and Adrenomedullin (ADM) have been widely used, but even these have limited capacity to distinguish sepsis from other inflammatory conditions, or to predict clinical outcomes. New biomarkers related to infectious diseases have been tested during recent years, but few has overcome the rigorous testing required for use in clinical practice [3–5].

DcR3 is a decoy receptor belonging to the tumour necrosis factor receptor super family (TNFRSF). It has three ligands: FasL, LIGHT and TL1A. By binding to these ligands, DcR3 can prevent interaction with their cell surface-bound receptors and thereby inhibits cell apoptosis [6]. We previously showed that the expression level of DcR3 are increased in serum of sepsis patients, and is associated with the severity of the disease. It demonstrated that DcR3 has potential value for differential diagnosis of sepsis and systemic inflammatory response syndrome (SIRS) [7]. We also reported that treatment targeted Decoy Receptor 3 (DcR3) can protect mice from sepsis by suppressing the inflammatory response and lymphocyte-associated apoptosis [8].

However, the mechanism by which DcR3 is up-regulated in sepsis patients remains unknown. Pathogens, via their microbial-associated molecular patterns, lipopolysaccharide (LPS, Gram-negative bacteria), lipoteichoic acid (LTA, Gram-positive bacteria) and zymosan (Fungus), trigger sequential intracellular events in immune cells, epithelium, and the neuroendocrine system. TLRs (Toll-like receptors) with an extracellular domain that participates in bacterial ligand recognition are the most widely described PRRs [9–12]. TLR-2 andTLR-4, which are expressed on the cell surface, are the only TLRs known to be responsive to microbial ligands [13]. TLR-2 and TLR-4 signalling pathways are fundamental in sepsis pathophysiology [14]. In this study, we explored DcR3 expression levels in human umbilical vein endothelial cells (HUVECs) and Jurkat cells stimulated by LPS, LTA and zymosan. SP600125, SB203580 and ammonium pyrrolidinedithiocarbamate (PDTC) were used to inhibit JNK1/2, p38MAPK and NF-κB signalling pathways respectively and study the signalling pathway regulating DcR3 secretion.

## Methods

### Clinic specimens collection

Sera from 30 normal adults and 36 sepsis patients whose personal identities had been removed were tested. This study was approved by the Ethics Committee of Songjiang District Center Hospital (Shanghai, China). The consent received from patinets was provided verbally. We notified them that we would collect the rest of blood samples after their clinical laborotary exmanition and detect DcR3 for data anlysis, which wouldn’t brought any clinical risk for them. The patients all agreed with the consent verbally. The Ethics Committee of Songjiang District Center Hospital approved this method of obtaining consent. The 30 healthy volunteers were age-matched with patients whose sera were tested in this study. Clinical and laboratory data of patients including age, gender, IL-6, CRP, PCT and APACHE II score were collected. Serum samples from sepsis patients were collected within 24 h after admission to ICU. After clotting, blood samples were centrifuged at 1200 g for 10 min, and serum was transferred into a clear tube and frozen at − 80 °C until needed.

### Cell culture and stimulations

HUVECs (ATCC) were cultured in Dulbecco’s modified Eagle’s medium (DMEM; Thermo Fisher Scientific, USA) supplemented with 10% fetal bovine serum (FBS) (Thermo Fisher Scientific, USA) and 1% penicillin/streptomycin (Thermo Fisher Scientific, USA) in an atmosphere of 5% CO_2_ at 37 °C. Cells were divided into four treatment groups: control, LPS (10 μg/mL), LTA (500 ng/mL) and zymosan (1000 μg/mL) (Sigma, St. Louis, MO, USA). Cells were planted at a density of 5 × 10^5^ cells/mL in six-well culture plates and cultured in DMEM medium without FBS for 12 h before addition of LPS, LTA or zymosan. DcR3 mRNA expression levels were detected at 12 h and protein was assayed at 24 h. SP600125, SB203580 and ammonium pyrrolidinedithiocarbamate (PDTC; all Sigma, St. Louis, MO, USA) were used to inhibit JNK1/2, p38MAPK and NF-κB signalling pathways respectively, at concentration of 25 μM, 20 μM and 100 μM. Inhibitors were added to the HUVECs cells 30 min before stimulation by LPS, LTA or Zymosan.

### Total RNA isolation and reverse transcription-PCR (RT-PCR) assays

Total RNA was extracted using an RNeasy kit (QIAGEN, USA) and quantified at 260 nm with a NanoDrop 2000 spectrophotometer (Thermo Scientific). Total RNA (1 μg) was reverse-transcribed to complementary DNA (cDNA) using an RT kit (Thermo Fisher Scientific). Quantitative PCR was performed in triplicate using a QuantiNova SYBR Green PCR Kit (QIAGEN, USA) on a ABI 7500 detection system (ABI, USA). The following primer sequences were used for amplification: *DcR3* forward 5′- CCACTACACGCAGTTCTGGA-3′, and reverse 5’-GTGCTCCAAGCAGAAACCAG- 3′, *β-actin* forward 5’-CCTGGCACCCAGCACAAT-3′ and reverse 5’-GGGCCGGACTCGTCATAC-3′. *β-actin* was used as the endogenous control and the comparative threshold cycle (2^-ΔΔCT^) equation was used to calculate the relative expression levels.

### ELISA and Western-blots

Changes in DcR3 levels in cell supernatants and sera were determined using an ELISA kit (RayBio, Norcross, GA, USA). Briefly, ELISA was performed in flat-bottomed 96-well plates that permitted the acquisition of data in a high throughput manner. Supernatants were incubated in wells for 2 h at room temperature then removed, and wells were washed with a series of buffer rinses. Biotin-labelled antibody was then added and incubated for 1 h at room temperature. The plate was washed three times and avidin-peroxidase was added for 30 min. Finally, addition of the enzyme substrate and measurement of colour production in wells was performed. The optical density (OD) value was determined at 595 nm.

Changes in expression of DcR3 in HUVECs and Jurkat cells were measured by western-blots. Briefly, cells were washed twice with ice-cold PBS and ruptured with lysis buffer. Protein concentration was determined by Bradford assay (Bio-Rad, Hercules, CA, USA). Equal quantities of protein were separated electrophoretically on 10% SDS-polyacrylamide gels and transferred onto polyvinylidene difluoride membranes (Roche, Basle, Switzerland). After blocking with 5% milk, the membrane was stained with the DcR3 primary antibody (CST No.4758, Boston, MA, USA) followed by HRP-conjugated secondary antibody (CST, Boston, MA, USA). Specific bands were visualized with an ECL Plus western blotting detection system (Bio-Rad). The gamma ratio value was calculated using Image Lab 6.0 software (Bio-Rad) and images from a ChemiDoc XRS+ system (Bio-Rad). The optical density of imaged bands was normalized against the GAPDH signal on the same blot. Three independent experiments were performed to obtain the mean ± SD.

### Flow cytometry

Expression of toll-like receptor 2 (TLR-2) and TLR-4 in HUVECs and Jurkat cells was determined by flow cytometry. Cells were seeded in six-well plates at a density of 1 × 10^5^ cells/well and incubated for 24 h. Following removal of the medium, cells were washed twice in ice-cold PBS, detached using trypsin and re-suspended in 500 μL of PBS. Jurkat cells were washed twice in ice-cold PBS by centrifuging at 1500 rpm for 5 min. PE-labelled TLR-2 (ab171568, abcam, Cambrisge, MA, USA) and FITC-labelled TLR-4 (ab8378, abcam, Cambrisge, MA, USA) primary antibodies were added to the cell resuspension solution at a dilution of 1:1000. After incubation for 30 min at room temperature, cells were washed twice in ice-cold PBS then immediately analysed by bivariate flow cytometry using FACScan (BD Biosciences, Franklin Lakes, NJ, USA) and Cell-Quest software (BD Biosciences). These experiments were performed at least three biological replicates.

### Statistical analysis

Data were analyzed using SPSS20.0 software (SPSS, Inc., Chicago, IL, USA). Parametric tests were carried out for normally distributed data, whereas non-parametric tests were used for skewed data. Comparisons between different treatments were made using one-way ANOVA, followed by either Tukey’s or Dunn’s post hoc test. Statistical significance was defined as *p* < 0.05. For quantitative variables, the mean ± SD is reported.

## Results

### DcR3 is up-regulated in sepsis patients

**Fig. 1:**
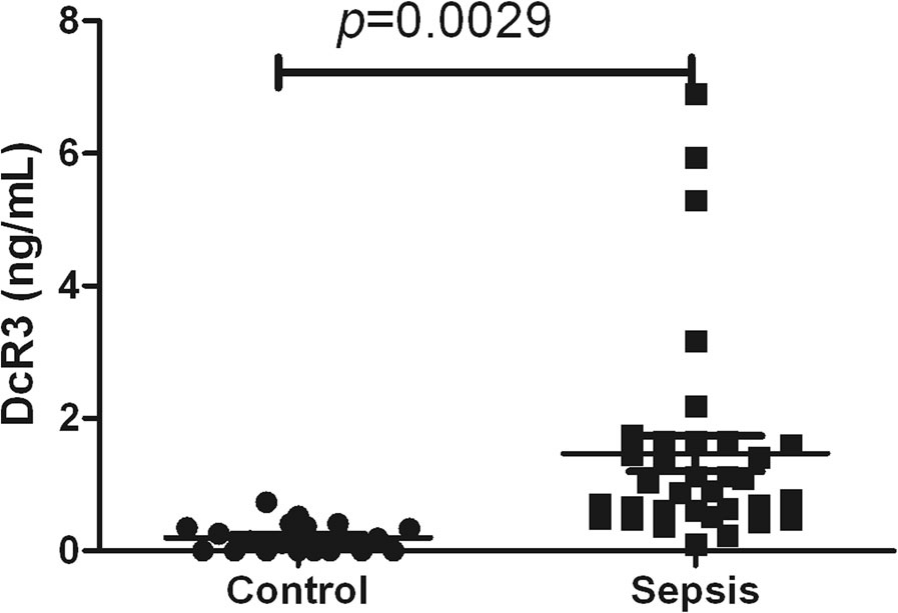
DcR3 is up-regulated in sepsis. DcR3 levels were higher in the sepsis group (1.44 ± 0.21 ng/mL) than the control group (0.22 ± 0.11 ng/mL; *p* = 0.0029)

DcR3 expression levels were higher in the sepsis patients group (1.44 ± 0.21 ng/mL) than the control group (0.22 ± 0.11 ng/mL, Fig. 1, *p* = 0.0029). The concentrations of IL-6 (100.45 ± 21.06 pg/mL), PCT (5.38 ± 0.58 ng/mL) and CRP (51.65 ± 6.25 ng/mL) were also higher in sepsis patients group. APACHE II score represents the severity of sepsis. It was shown DcR3 had a close correlation with APACHE II score. Details of patients and controls are shown in Table 1.

**Table 1:** Clinical data of sepsis patients

	Control (*n* = 30)	Sepsis (*n* = 36)
Age	59.65 ± 11.04	65.46 ± 13.02
M/F	10/20	11/25
DcR3 (ng/mL)	0.22 ± 0.11	1.44 ± 0.21^a^
IL-6 (pg/mL)	6.45 ± 1.21	100.45 ± 21.06 ^a^
PCT (ng/mL)	0.06 ± 0.002	5.38 ± 0.58 ^a^
CRP (ng/mL)	4.65 ± 0.45	51.65 ± 6.25 ^a^
APACHEII	–	23.01 ± 1.02

^a^Sepsis vs. Control, *p* < 0.05

### DcR3 is up-regulated at both mRNA and protein levels following stimulation with LPS, LTA or Zymosan

**Fig. 2:**
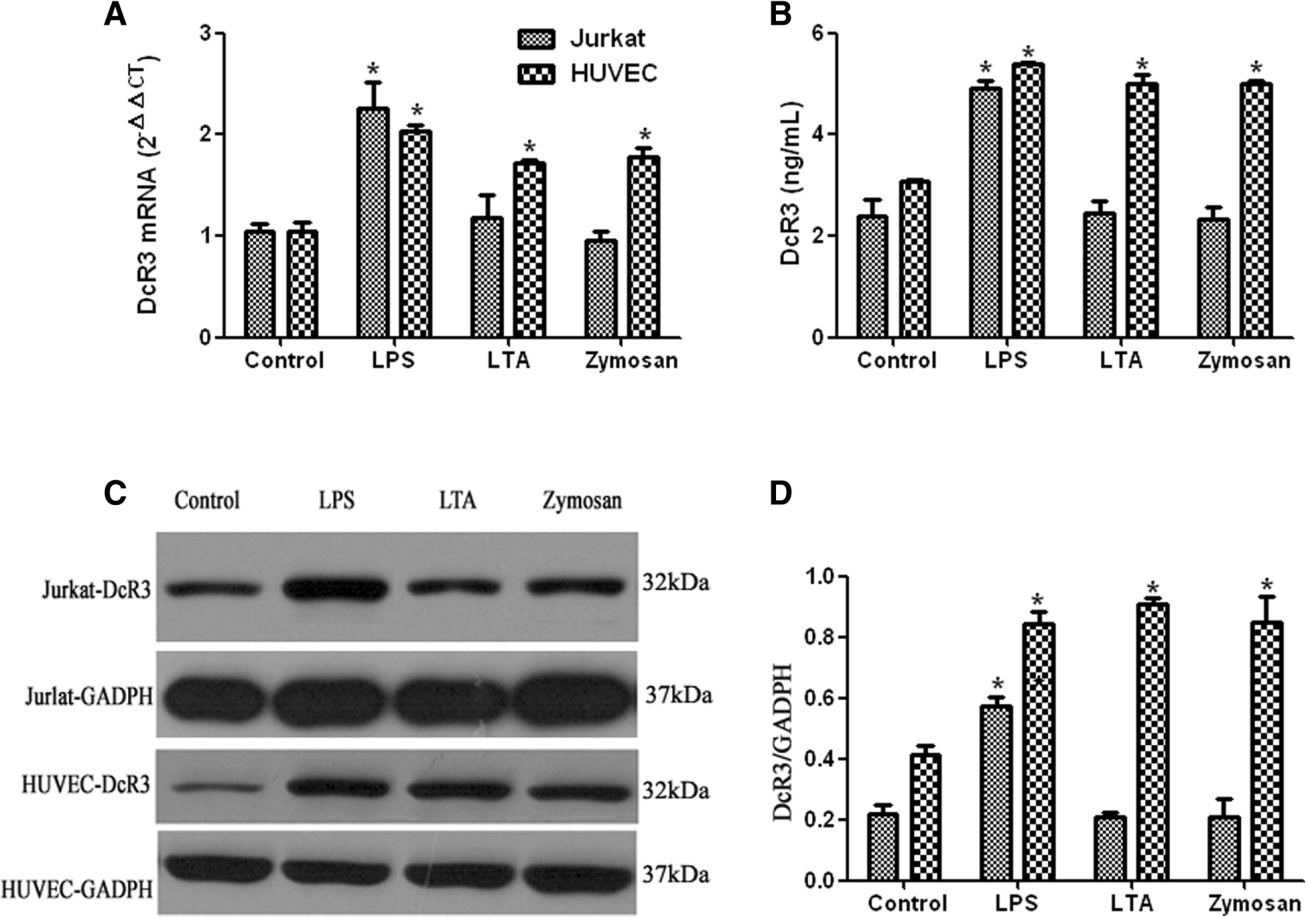
DcR3 changes at both mRNA and protein levels following stimulation with LPS, LTA or Zymosan. DcR3 in HUVECs were increased following treatment with10 μg/mL LPS, 500 ng/mL LTA and 1000 μg/mL zymosan for 12 h, compared with controls. DcR3 mRNA levels in Jurkat cells were also increased following treatment with 10 μg/mL LPS for 12 h, compared with controls, but not following treatment with LTA or zymosan. **a** DcR3 mRNA changes by real-time PCR. **b** DcR3 changes by ELISA. **c** and **d** DcR3 changes by western blotting. *compared with control

The relative mRNA levels of DcR3 in HUVECs were increased following treatment with 10 μg/mL LPS (2.03 ± 0.07), 500 ng/mL LTA (1.71 ± 0.04) and 1000 μg/mL zymosan (1.77 ± 0.09) for 12 h, compared with control group (1.04 ± 0.10; Fig. 2a). DcR3 mRNA expression level in Jurkat cells were also increased following treatment with 10 μg/mL LPS (2.26 ± 0.26) for 12 h, compared with controls (1.04 ± 0.08), but not following treatment with LTA (1.18 ± 0.22) or zymosan (0.96 ± 0.09; Fig. 2a). ELISA and western blotting confirmed the same patterns in DcR3 protein levels in HUVECs and Jurkat cells co-incubated with LPS, LTA or Zymosan for 24 h (Fig. 2b, c, d).

### Toll-like receptor expression in HUVECs and Jurkat cells

**Fig. 3:**
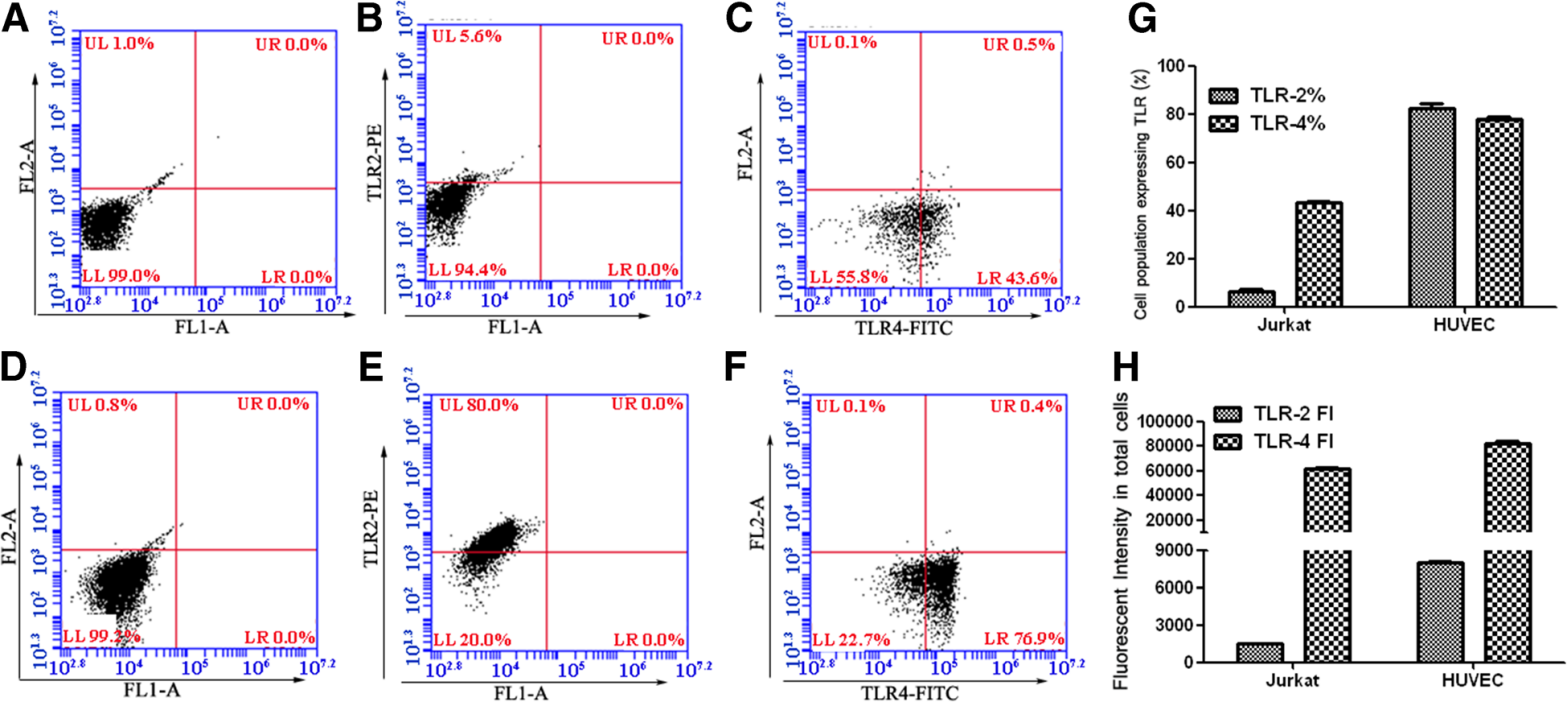
Toll-like receptor expression in HUVECs and Jurkat cells. TLR-2 and TLR-4 receptor expression was assayed using flow cytometry. Expression of both TLR-2 and TLR-4 was in HUVECs; however, TLR-4 expression was significantly higher than TLR-2 expression in Jurkat cells. **a** Jurkat cells were assayed by flow cytometry without antibody incubation. **b**, **c** Jurkat cells were treated by PE-labbled TLR-2 primary antibody and FITC-labbled TLR-4 primary antibody separately and then assayed the TLRs expression by flow cytometry. **d** HUVECs were assayed by flow cytometry without antibody incubation. **e**, **f** HUVECs cells were treated by PE-labbled TLR-2 primary antibody and FITC-labbled TLR-4 primary antibody separately and then assayed the TLRs expression by flow cytometry. **g** The TLR-2 and TLR-4 expression ratio in Jurkat cells and HUVECs. **h** The TLR-2 and TLR-4 fluorescence intensity in Jurkat cells and HUVECs

TLR-2 and TLR-4 receptor expression was examined using flow cytometry. The TLR-2 and TLR-4 expression ratio in HUVECs was 82.20 ± 2.15% and 77.86 ± 1.86%, compared with 6.3 ± 0.96% and 43.10 ± 0.78% in Jurkat cells. The TLR-2 and TLR-4 fluorescence intensity in HUVECs was 8019.79 ± 144.30 and 82,632.22 ± 3258.04, compared with 1525.90 ± 77.78 and 62,127.14 ± 984.72 in Jurkat cells. Expression of TLR-2 and TLR-4 was therefore clearly higher in HUVECs; however, TLR-4 expression was significantly higher than TLR-2 expression in Jurkat cells (*p* < 0.05, Fig. 3).

### DcR3 secretion in HUVECs is regulated by the NF-κB signalling pathway

**Fig. 4:**
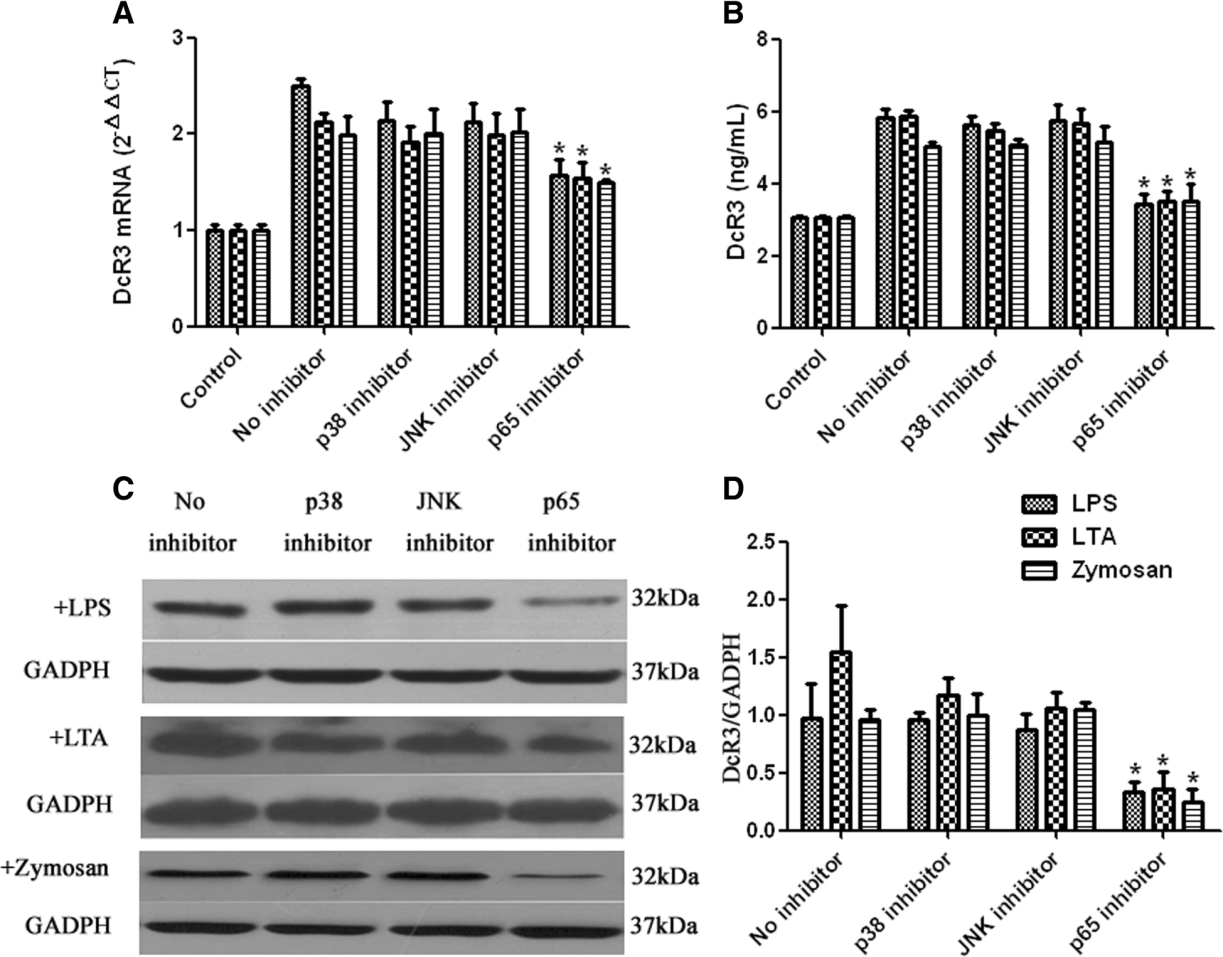
DcR3 secretion in HUVECs is regulated by the NF-κB signalling pathway. P38MAPK inhibitor SB203580, JNK1/2 inhibitor SP600125 and NF-κB inhibitor PDTC were used to study the pathways involved inDcR3 secretion. DcR3 transcription and expression was inhibited by PDTC but not by SB203580 or SP600125, compared with control cells not treated with inhibitors. **a** DcR3 mRNA changes by real-time PCR. **b** DcR3 changes by ELISA. **c** and **d** DcR3 changes by western blotting. *compared with No inhibitor groups

**Fig. 5:**
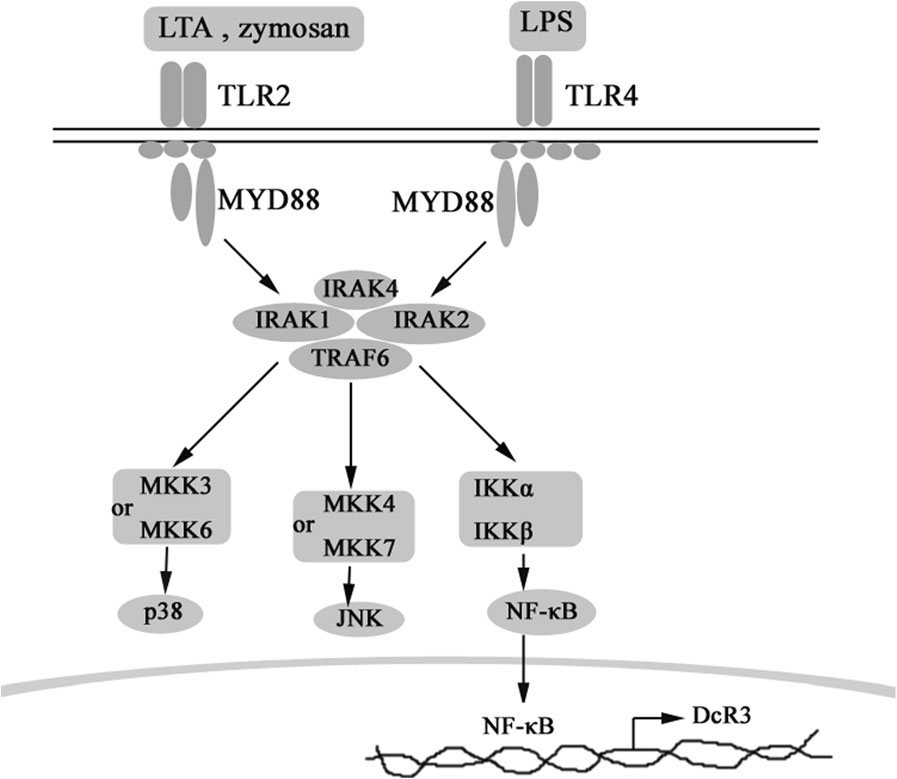
NF-κB signalling pathway is involved in endogenous DcR3 expression in HUVECs stimulated by LPS, LTA and zymosan. TLR-2 and TLR-4 utilize MyD88-adaptor-like (MAL), which lead to the activation of MAPKs and NF-κB. The active NF-κB promotes DcR3 secretion

P38MAPK inhibitor SB203580, JNK1/2 inhibitor SP600125 and NF-κB inhibitor PDTC were used to study the pathways involved in DcR3 secretion. HUVECs were stimulated by LPS, LTA and zymosan after the treatments of these inhibitors, and comparative DcR3 mRNA analysis showed that DcR3 transcription was inhibited by PDTC (1.57 ± 0.17) but not by SB203580 (2.13 ± 0.20) or SP600125(2.12 ± 0.20), compared with control cells no treated with inhibitors (2.50 ± 0.08). Furthermore, ELISA and western-blot assays confirmed the same patterns in DcR3 protein levels (inhibition by PDTC but not SB203580 or SP600125 (Fig. 4). These results demonstrated that the NF-κB signalling pathway is involved in endogenous DcR3 expression in HUVECs stimulated by LPS, LTA and zymosan (Fig. 5).

## Discussion

Decoy receptor 3 (DcR3/TR6) is a newly identified member of the decoy receptor family. This secreted protein belongs to the family of tumour necrosis factor receptor. DcR3 has three main ligands: FasL, LIGHT and TL1A. DcR3 binding to FasL protects against FasL-mediated apoptosis of lymphocytes and various tumour cell types, whereas DcR3 binding to LIGHT inhibits LIGHT-induced apoptosis [15–17]. Meanwhile, DcR3 binding to TL1A induces T cell activation [18]. Expression of DcR3 has been demonstrated in various acute and chronic inflammatory conditions such as inflammatory bowel diseases (IBD) and acute respiratory distress syndrome (ARDS) [19, 20]. DcR3 was expressed in THP-1 and increased by phorbol 12-myristate 13-acetate (PMA).The formation of macrophage aggregates was observed when THP-1 cells were stimulated with DcR3-Fc [21]. Yang showed that pretreatment of HUVECs with DcR3 enhances the adhesion of THP-1 and U937 cells and primary monocytes [22]. DcR3 regulates CD14+ monocyte differentiation into dendritic cells. In addition, DcR3 triggers actin re-organization, increases the adhesion of monocytes, and reduces phagocytic activity and proinflammatory cytokine production in macrophages [23].

Our previous study shown that DcR3 is up-regulated in sepsis patients and levels are correlated with disease severity. However, the mechanism of elevated DcR3 in sepsis remains unknown. In this study, we stimulated HUVECs and Jurkat cells with LPS (Gram-negative bacteria), LTA (Gram-positive bacteria) or zymosan (fungi) to investigate DcR3 expression levels and secretion pathways. A previous study showed DcR3 was able to induce angiogenesis in human umbilical vein endothelial cells (HUVECs) but not in human aortic endothelial cells (HAECs) and DcR3 was higher expressed in HUVECs than in HAECs, which indicated the angiogenic action of DcR3 is endothelial cell type specific [24].

The majority of sepsis cases involve infection with Gram-positive or Gram-negative bacteria, or fungi [25, 26]. The innate immune system forms the first line of defence against microbial infection and is activated by the engagement of innate immune receptors, also known as pattern recognition receptors (PRRs), in response to invading pathogenic microbes [27–29].

TLR-4 associates with the adapter protein MD2 during recognition of LPS, which initiates downstream signalling events. Meanwhile, TLR-2 recognizes a broad range of microbial and host inflammatory agonists [30, 31]. Inflammation mediated by endothelial cells and inflammatory cell plays an important role in the process of sepsis. Endothelial cells dynamically regulate the vascular barrier, modulate vasomotor tone, play central roles in coagulation and hemostasis, and are critically involved in the movement of leukocytes between the bloodstream and extravascular tissues. Although a great deal is known about the effects of microorganisms on monocyte and macrophage inflammatory pathways, substantially less is known about their effects on endothelial cells, which have not classically been viewed as immune cells. However, similar to leukocytes, endothelial cells express innate immune receptors, including members of the TLRs family [32]. Flow cytometry experiments showed that TLR-2 and TLR-4 were highly expressed in HUVECs, while Jurkat cells displayed high TLR-4 expression but much lower TLR-2 expression. In the present study, we imitated sepsis using a cellular model by exposing cells to LPS (Gram-negative bacteria), LTA (Gram-positive bacteria) and zymosan (fungi) to stimulate HUVECs or Jurkat cells.

We want to know whether the high DcR3 levels in sepsis was due to the secretion by HUVECs or Jurkat cells. In the present study, DcR3 was induced by LPS, LTA or zymosan in HUVECs, and it was also up-regulated in Jurkat cells by LPS. Jurkat cells displayed relatively lower expression TLR-2, which is known to recognize LTA and zymosan. This could explain why stimulation of Jurkat cells by LTA or zymosan did not increase DcR3 expression. These results indicated that the high DcR3 concentration in sepsis patients was due to HUVECs and Jurkat cells stimulated by bacterial toxin.

To improve the clinical management of and outcomes for critically ill patients, Surviving Sepsis Campaign guidelines have been published and revised [33]. However, to date, there have been no commercially available successful strategies for targeting the molecular pathways involved in sepsis. In this study, we used pathway inhibitors to investigate DcR3 expression secretion signalling.

1TLR-2 and TLR-4 utilize the adaptor TIR domain-containing adapter protein (TIRAP), also known as MyD88-adaptor-like (MAL), which lead to the activation of MAPKs and NF-κB [34–37]. Conventional MAPK family members include p38-MAPK, c-Jun N-terminal kinase, extracellular-signal-regulated kinase (ERK)-1/2 and ERK5. The role of ERK1/2 signalling downstream of TLRs is complex, and studies to define the role of ERK1/2 have been hampered by the fact that several commonly used pharmacological inhibitors of MEK1, the upstream kinase of ERK1/2, inhibit the activity of both MEK1 and MEK5, the upstream kinase of ERK5 (e.g. PD98059, U0126 and PD184352 at concentrations > 1 mM). Therefore, it is not always clear whether the observed outcomes are due to the loss of ERK1/2 or ERK5 kinase activity. ERK5 is a recently identified mediator of the inflammatory response, but the signalling pathways that lead to the activation of ERK5 downstream of TLRs are not known [38–42].

Despite this complexity, we are relatively confident of three definite TLR-2 and TLR-4 common signalling pathways: TLR2/TLR4-MyD88/IRAK-NF-κB, TLR2/TLR4-MyD88/IRAK-p38 and TLR2/TLR4-MyD88/IRAK-JNK. Notably, endothelial cells such as leukocytes express the intermediary TLR signalling components that are required for activation of the MAPKs and NF-κB pathways. Because we showed that HUVECs express both TLR-2 and TLR-4 receptors at high levels, we used HUVECs to study the signalling pathways involved in DcR3 secretion, and used NF-κB, p38 and JNK signalling pathway inhibitors to study the mechanism of DcR3 secretion in HUVECs stimulated by LPS, LTA or zymosan. The results showed that DcR3 was secreted in HUVECs through the NF-κB pathway.

Transcription factor NF-κB participates in cell proliferation, apoptosis, immune inflammation and tumorigenesis, and activation of NF-κB reportedly promotes the expression of DcR3. Xia et al. (2012) found that when the human pancreas cell line ASPC-1 was challenged with LPS or TNF-α, the active NF-κB up-regulated DcR3 expression. Meanwhile, Chen and Yang (2008) found that human intestinal epithelial cells secreted DcR3 through TLR/PI3K/NF-κB regulation, and Ho et al. (2007) found that the EB virus promoted DcR3 expression by activating NF-κB [43–45]. The NF-κB pathway was critically involved in the molecular mechanisms underlying the action of EGFR and inflammatory cytokines [46]. In our present research, we also found that DcR3 was up-regulated via the NF-κB pathway in the sepsis cell models.

## Conclusions

Combined with our previous studies, our results showed that DcR3 expression is increased in sepsis patients, and DcR3levelsare correlated with the severity of the disease. In cecal ligation and puncture (CLP)-induced sepsis mouse models, DcR3 protein treatments markedly improved survival in septic mice, reduced the inflammatory response, and decreased lymphocyte apoptosis in the thymus and spleen [8]. In the present study, we found that elevated DcR3 levels in septic cell models proceeded through the NF-κB signalling pathway, as did DcR3 secretion in HUVECs stimulated by LPS, LTA or zymosan. Taken together, the emerging evidence suggests that DcR3 plays an important role in sepsis and may be a therapeutic target for sepsis treatment.

## Abbreviations

ADM: Adrenomedullin
ARDS: acute respiratory distress syndrome
CRP: C-reactive protein
DcR3: Decoy Receptor 3
HAECs: Human aortic endothelial cells
HUVECs: Human umbilical vein endothelial cells
IBD: Inflammatory bowel diseases
IL-18: Interleukin 18
IL-6: Interleukin 6
IL-8: Interleukin 8
LPS: Lipopolysaccharide
LTA: Lipoteichoic acid
NGAL: Human neutrophil gelatinase
PCT: Procalcitonin
PDTC: Pyrrolidinedithiocarbamate
PMA: Phorbol 12-myristate 13-acetate
PRRs: Pattern recognition receptors
SIRS: Systemic inflammatory response syndrome
TNFRSF: Tumour necrosis factor receptor super family

## References

[1] Stevenson M, Pandor A, Martyn-St James M, Rafia R (2016). Sepsis: the Light cycler SeptiFast test MGRADE®, SepsiTest™ and IRIDICA BAC BSI assay for rapidly identifying bloodstream bacteria and fungi - a systematic review and economic evaluation. Health Technol Assess.

[2] Lanziotti VS, Póvoa P, Soares M (2016). Use of biomarkers in pediatric sepsis: literature review. Rev Bras Ter Intensiva.

[3] Heffner A, Mahapatra S (2017). Shock, Septic (Sepsis). StatPearls. Treasure Island (FL): StatPearls publishing.

[4] Prucha M, Bellingan G, Zazula R (2015). Sepsis biomarkers. ClinChimActa.

[5] Dupuy AM, Philioart F, Pean Y, Lasocki S, Charles PE (2013). Role of biomarkers in the management of antibiotic therapy: an expert panel review: I currently available biomarkers for clinical use in acute infections. Ann Intensive Care.

[6] Lin WW, Hsieh SL (2011). Decoy receptor 3: a pleiotropic immunomodulator and biomarker for inflammatory diseases, autoimmune diseases and cancer. Biochem Pharmacol.

[7] Hou YQ, Xu P, Zhang M, Han D, Peng L (2012). Serum decoy receptor 3, a potential new biomarker for sepsis. ClinChimActa..

[8] Liang D, Hou Y, Lou X, Chen H (2015). Decoy receptor 3 improves survival in experimental Sepsis by suppressing the inflammatory response and lymphocyte apoptosis. PLoS One.

[9] Shibata T, Nakashima F, Honda K, Lu YJ, Kondo T (2014). Toll-like receptors as a target of food-derived anti-inflammatory compounds. J Biol Chem.

[10] Hoang M, Potter JA, Gysler SM, Han CS, Guller S (2014). Human fetal membranes generate distinct cytokine profiles in response to bacterial toll-like receptor and nod-like receptor agonists. Biol Reprod.

[11] Beutler BA (2009). TLRs and innate immunity. Blood.

[12] Foley NM, Wang J, Redmond HP, Wang JH. Current knowledge and future directions of TLR and NOD signaling in sepsis. Mil Med Res. 2015;2(1). 10.1186/s40779-014-0029-7 PMC4340879 25722880

[13] Brightbill HD, Modlin RL (2000). Toll-like receptors: molecular mechanisms of themammalian immune response. Immunology.

[14] Bosmann M, Ward PA (2013). The inflammatory response in sepsis. Trends Immunol.

[15] Toda M, Kawamot T, Ueha T, Kishimoto K, Hara H (2013). ‘Decoy’ and ‘non-decoy’ functions of DcR3 promotemalignant potential in humanmalignant fibrous histiocytoma cells. Int J Oncol.

[16] Chen L, Tian X, Li W, Agarwal A, Zhuang G (2009). Expressions of Fas/DcR3 and RGD-FasLmediatedapo-ptosis in pituitary adenomas. Neurol India.

[17] Hsieh SL, Lin WW (2017). Decoy receptor 3: an endogenous immunomodulator in cancer growth and inflammatory reactions. J Biomed Sci.

[18] Lee CS, Hu CY, Tsai HF, Wu CS, Hsieh SL (2008). Elevated serumdecoy receptor 3 with enhanced T cell activation in systemic lupus erythematosus. ClinExpImmunol.

[19] Chen CY, Yang KY, Chen MY, Chen HY, Lin MT (2009). Decoy receptor 3 levels in peripheral blood predict outcomes of acute respiratory distresssyndrome. Am J RespirCrit Care Med.

[20] Cardinale CJ, Wei Z, Panossian S, Wang F, Kim CE (2013). Targeted resequencing identifies defective variants of decoy receptor 3 in pediatric-onset inflammatory bowel disease. Genes Immun.

[21] Tateishi K, Miura Y, Hayashi S, Takahashi M, Kurosaka M (2009). DcR3 protects THP-1 macrophages from apoptosis by increasing integrin alpha4. Biochem Biophys Res Commun.

[22] Yang CR, Hsieh SL, Ho FM, Lin WW (2005). Decoy receptor 3 increases monocyte adhesion to endothelial cells via NF-kappa B-dependent up-regulation of intercellular adhesion molecule-1, VCAM-1, and IL-8 expression. J Immunol.

[23] Wu YY, Chang YC, Hsu TL, Hsieh SL, Lai MZ (2004). Sensitization of cells to TRAIL-induced apoptosis by decoy receptor 3. J Biol Chem.

[24] Yang CR, Hsieh SL, Teng CM, Ho FM, Su WL, Lin WW (2004). Soluble decoy receptor 3 induces angiogenesis by neutralization of TL1A, a cytokine belonging to tumor necrosis factor superfamily and exhibiting angiostatic action. Cancer Res.

[25] Yao YM, Luan YY, Zhang QH, Sheng ZY (2015). Pathophysiological aspects of sepsis: an overview. Methods Mol Bio.

[26] Balk RA (2014). Systemic inflammatory response syndrome (SIRS): where did it come fromand is it still rele-vant today?. Virulence.

[27] Ishii KJ, Koyama S, Nakagawa A, Coban C, Akira S (2008). Host innate immune receptors and beyond: mak-ing sense of microbial infections. Cell Host Microbe.

[28] Akira S, Uematsu S, Takeuchi O (2006). Pathogen recognition and innate immunity. Cell.

[29] Sellge G, Kufer TA (2015). PRR-signaling pathways: learning from microbial tactics. Semin Immunol.

[30] Da Silva CJ, Soldau K, Christen U, Tobias PS, Ulevitch RJ (2001). Lipopolysaccharide is in close proximity to each of the proteins in its membrane receptor complex. Transfer from CD14 to TLR4 and MD-2. JBiolChem.

[31] Bulut Y, Faure E, Thomas L, Karahashi H, Michelsen KS (2002). Chlamydial heat shock protein 60 activates macrophages and endothelial cells through toll-like receptor 4 and MD2 in a MyD88-dependent pathway. J Immunol.

[32] Khakpour S, Wilhelmsen K, Hellman J (2015). Vascular endothelial cell toll-like receptor pathways in sepsis. Innate Immunity.

[33] Schorr CA, Dellinger RP (2014). The surviving Sepsis campaign: past, present and future. Trends Mol Med.

[34] Horng T, Barton GM, Flavell RA, Medzhitov R (2002). The adaptor molecule TIRAP provides signalling specificity for toll-like receptors. Nature.

[35] Yamamoto M, Sato S, Hemmi H, Sanjo H, Uematsu S (2002). Essential role for TIRAP in activation of the signalling cascade shared by TLR2 and TLR4. Nature.

[36] Arthur JS, Ley SC (2013). Mitogen-activated protein kinases in innate immunity. Nat Rev Immunol.

[37] Ye X, Ding J, Zhou X, Chen G, Liu SF (2008). Divergent roles of endothelial NF-kappaB in multiple organ injury and bacterial clearance in mouse models of sepsis. J Exp Med.

[38] Cargnello M, Roux PP (2011). Activation and function of the MAPKs and their substrates, the MAPK-activated protein kin-ases. Microbiol Mol Biol Rev.

[39] Shaul YD, Seger R (2007). The MEK/ERK cascade: from signaling specificity to diverse functions. Biochem Biophys Acta.

[40] Glatz G, Gogl G, Alexa A, Reményi A (2013). Structural mechanism for the specific assembly and activation of the extracellular signal regu-lated kinase 5 (ERK5) module. J Biol Chem.

[41] Bain J, Plater L, Elliott M, Shpiro N, Hastie CJ (2007). The selectivity of protein kinase inhibitors: a further update. Biochem J.

[42] Mody N, Leitch J, Armstrong C, Dixon J, Cohen P (2001). Effects of MAP kinase cascade inhibitors on the MKK5/ERK5 pathway. FEBS Lett.

[43] Ho CH, Hsu CF, Fong PF, Tai SK, Hsieh SL (2007). Epstein-Barr virus transcription activator Rtaupregulates decoy receptor 3 expression by binding to its promoter. J Virol.

[44] Xia LM, Tian DA, Huang WJ, Zhu H, Wang J (2012). Upregulation of IL-23 expression in patients with chronic hepatitis B is mediated by the HBx/ERK/NF-kappa B pathway. J Immunol.

[45] Chen P, Yang C (2008). Decoy receptor 3 expression in AsPC-1 human pancreatic adenocarcinoma cells via the phosphatidylinositol 3kinase,Akt,and NF-kappa B-dependent pathway. J Immunol.

[46] Wu NL, Huang DY, Hsieh SL, Hsiao CH, Lee TA, Lin WW (2013). EGFR-driven up-regulation of decoy receptor 3 in keratinocytes contributes to the pathogenesis of psoriasis. Biochim Biophys Acta.

